# An Unusual Case of Hand Xanthomatosis

**DOI:** 10.1155/2013/183018

**Published:** 2013-06-04

**Authors:** Gazi Huri, Noah Joachim

**Affiliations:** ^1^Department of Orthopaedic and Traumatology Surgery, Balcali Hospital, Cukurova University, Adana 01030, Turkey; ^2^Division of Sport Medicine, Department of Orthopaedics and Traumatology Surgery, Johns Hopkins University, 10753 Falls Road, Suites 215, Lutherville, Baltimore, MD 21093, USA; ^3^Johns Hopkins University Zanvyl Krieger School of Arts and Sciences, Wyman Park Drive Baltimore, MD 21211, USA

## Abstract

Tendon xanthomatosis often accompanies familial hypercholesterolaemia, but it can also occur in other pathologic states. Of the musculoskeletal system, the Achilles tendon is the most commonly effected tendon due to xanthomatosis. Although there are previous reports for flexor tendon involvement, to our knowledge there is no report in the English literature about bilateral hand extensor tendon xanthomatosis that causes restriction in the range of motion. The case that will be presented in this report is therefore unique.

## 1. Introduction

Xanthomas are nonneoplastic tumors that represent a physical manifestation of hyperlipidemia. Particularly, tendon xanthomas often appear as a clinical manifestation of familial hypercholesterolemia (FH). FH affects approximately 1 in every 500 people and the incidence of tendon xanthomas in FH increases with age, as is reflected by 75% of heterozygotes [1]. High cholesterol presents a significant health risk in the United States, and although high cholesterol is a known risk factor for heart disease and stroke, its potential role in the musculoskeletal system is not well understood. Therefore, because musculoskeletal symptoms (xanthomas) often precede the diagnosis of hyperlipidemia, it is important that orthopaedic surgeons are familiar with FH because patients with FH are at high risk for premature coronary atherosclerosis [1].

 The most common involvements of xanthomas in orthopaedics include the Achilles tendons and hands [2]. Specifically dealing with hand involvement, it is known that xanthomas can be present in flexor and extensor tendons. Reports in the hand surgery literature, however, have been sparse. Although there are previous reports for flexor tendon involvement [3, 4], only three cases in the English language literature have concentrated on hand extensor tendon xanthomas [5]. Furthermore, the case that will be presented in this report is even more unique as symptoms are observed bilaterally with a restriction in extension.

## 2. Case Report

**Figure 1:**
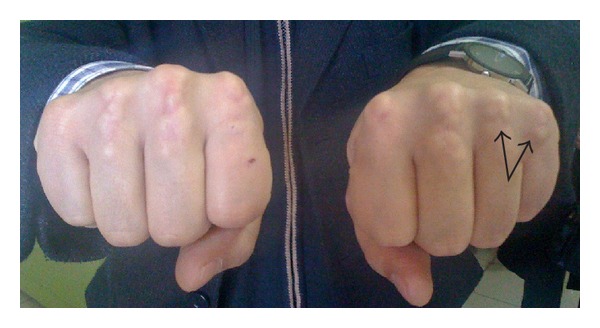
Bilateral hand extensor tendon masses around metacarpophalangeal joints (MCP) are clearly defined. Black arrows indicate the masses at the symptomatic joints.

**Figure 2:**
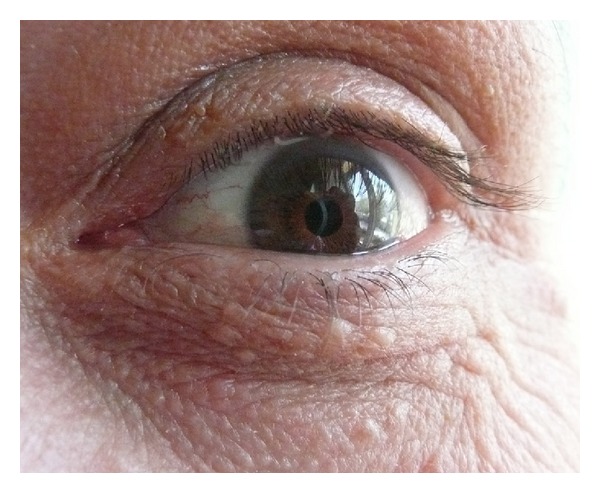
A grayish-white ring opacity occurring in the periphery of the cornea.

A 42-year-old man made a complaint of restriction of motion particularly in the 4th and 5th fingers of his left hand. He had never been examined before because the restriction of motion had only arisen in the past month. The painless masses were growing slowly and were causing dysfunction of the affected joints. Although all of the MCP joints were covered with these solid and painless masses (Figure 1), only the 4th and 5th MCP joints exhibited extension loss (20 and 30 degrees, resp., in the left hand). The neurovascular examination was intact. The patient has bilateral arcus cornea, which may be an indication of hypercholesterolemia (Figure 2). The patient's serum cholesterol level was above normal, and he has a positive family history of early death due to myocardial infarction. There were no abnormal findings observed in the plain X-rays; however, multiple solid masses that were engaged to the extensor tendons were detected in the superficial ultrasound (USG). The severity of engagement was remarkable at the level of the MCP joints of the 4th and 5th fingers. Regarding our findings and the clinical progression, the patient underwent an open excisional biopsy and tenolysis of the extensor tendon at the 4th and 5th MCP joints.

**Figure 3:**
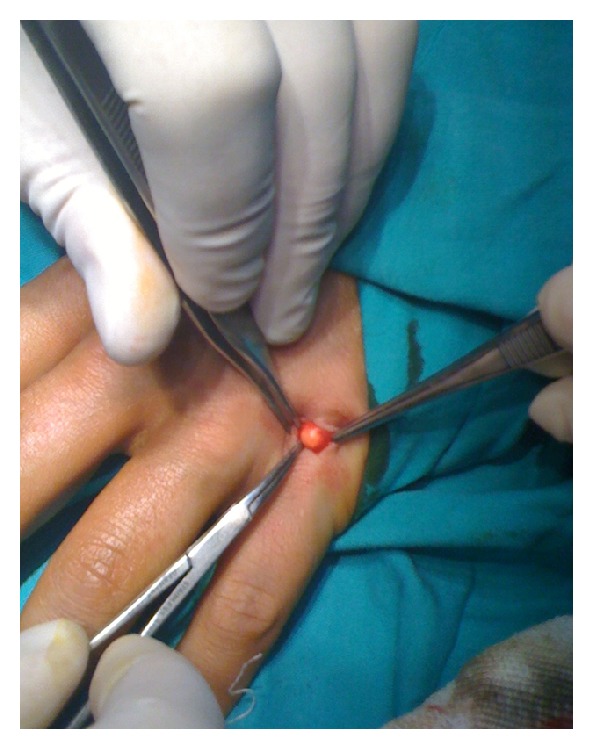
Intraoperative finding of the solid and yellowish mass at the extensor side of 5th metacarpophalangeal joint.

Under local anesthesia and tourniquet control, a “V-shaped” excision was used to explore the masses over the extensor side of the 4th and 5th MCP joints. After the retraction of the subcutaneous tissue, 2 × 2 cm and 3 × 2 cm, solid, yellowish masses were detected over the extensor side of the 4th and 5th MCP joints, respectively (Figure 3). Peritendinous fibrosis were observed as these masses infiltrated the extensor tendons thus restricting the gliding of the tendons. All masses were meticulously dissected from the tendon with devoted attention to maintain the continuity of the tendon by preserving the paratenon. The full range of motion was achieved after excision of the masses and the wound was sutured. Immediate active and passive ranges of motion exercises were started to avoid stiffness. Although the literature mentions wound complications, no issue with wound healing was encountered [1].

**Figure 4:**
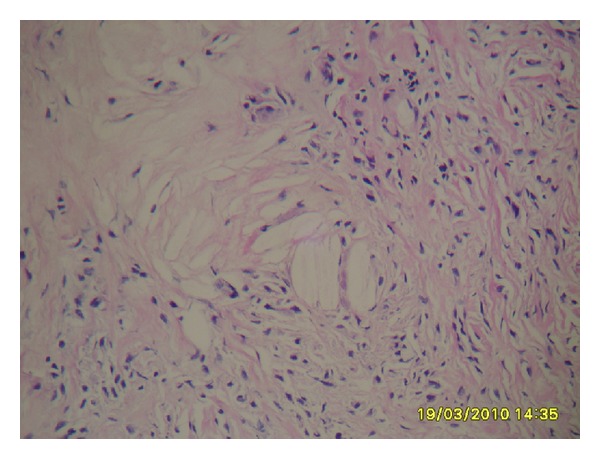
Microscopic examination revealed cholesterol clefts admixed with histiocytes and occasional giant cells in fibrous stroma suggesting a tendinous xanthoma (hematoxylin-eosin × 200).

A biopsy specimen showed xanthoma cells dispersed between the collagen fibers of the tendon (Figure 4). After thirty months followup, no recurrence or complications were detected in regard to maintaining a full range of motion of the affected joints.

## 3. Discussion

 To our knowledge, this case is the first case in the English literature regarding xanthoma leading to restrained motion of the joints. It is also the first case presenting bilateral involvement of the extensor tendons at the level of the MCP joints.

Xanthomas are considered as reactive lesions, associated with hyperlipidemia. Prominent fibrosis can occur in long standing lesions resulting from the fibrogenic properties of extracellular cholesterol. They are also defined by the deposition of yellowish cholesterol rich material in tendons or other body parts. The detection of xanthomas is critical to an early diagnosis of the disease, which is extremely important to be able to alter the course of the disease before the onset of coronary artery disease.

Regarding the orthopaedics practice, xanthomas are particularly seen around tendons such as Achilles, patellar tendon, elbow, and hand tendons [6, 7]. The tendons involved with those fatty deposits are biomechanically weaker and tend to be more susceptible to tendon injuries and ruptures [8]. They infiltrate the tendon as well as adjacent tissues and may lead to joint stiffness, as in our case [9].

Most cases of tendon xanthoma occur at the Achilles tendon, with only a few reports mentioning surgical treatment of hand xanthoma [10]. One particular case reported by Gunther et al. [3] presented a 6-year-old girl with familial hypercholesterolemia who had flexion contractures of the fingers due to tendon and joint xanthomas. Aside from cases involving the flexor tendon of the hand, Doyle reported a case of one patient treated by surgical excision of large xanthomas arising from the substance of the extensor tendons over MCP joints of the hand [6]. Our case, however, is unique from all other previously reported cases in that bilateral involvement of the extensor tendon is observed as well as a restriction in extension. Furthermore, this is the only case to be found where surgery took place on the extensor tendon not for mere cosmetic purposes, but for functional purposes as well.

Because these lesions arise from the substance of the extensor tendons, complete removal through surgery may result in loss of tendon continuity and function, as well as wound complications and/or joint stiffness. As a result, surgery is advised primarily for functional or cosmetic reasons. For all tendon involvements of xanthoma, the necessity of surgery is therefore dependent on the desires of the patient. This patient was operated on due to the patient's complaint of restriction of motion and thus a direct effect on functionality.

Differential diagnoses from giant cell tumor of the tendon sheet can be challenging [11]. In contrast to giant cell tumors of the tendon sheet, tendinous xanthomas are associated with hyperlipidemia and are usually numerous. Also microscopically tendinous xanthomas usually contain relatively few multinucleated giant cells compared to giant cell tumor of the tendon sheet. Additionally, tendinous xanthomas lack round cells, which is an important feature of giant cell tumor of the tendon sheet. Although tendinous xanthoma is believed to be a reactive lesion, sarcomas with xanthomatous changes may focally resemble tendinous xanthomas. Adequate sampling of the lesion is essential to ensure that tendinous xanthomas lack any atypical features of a sarcoma [12].

Among several clinical manifestations as mentioned above, xanthoma is also associated with a three times higher risk of cardiovascular disease among familial hypercholesterolemia patients, suggesting that xanthomas and cardiovascular disease share etiology [13]. In conclusion, the role of the orthopaedic surgeon to go beyond orthopaedics in order to recognize the much broader spectrum of risk xanthomas present to the given patient cannot be overemphasized.
